# Tunable Helmholtz Resonators Using Multiple Necks

**DOI:** 10.3390/mi14101932

**Published:** 2023-10-15

**Authors:** Nikolaos M. Papadakis, Georgios E. Stavroulakis

**Affiliations:** Institute of Computational Mechanics and Optimization (Co.Mec.O), School of Production Engineering and Management, Technical University of Crete, 73100 Chania, Greece; gestavroulakis@tuc.gr

**Keywords:** Helmholtz resonator, multi-neck Helmholtz resonator, sound absorber, architectural acoustics, room acoustics, musical acoustics, finite element method, sound absorption, acoustic transmission

## Abstract

One of the uses of Helmholtz resonators is as sound absorbers for room acoustic applications, especially for the low frequency range. Their efficiency is centered around their resonance frequency which mainly depends on elements of their geometry such as the resonator volume and neck dimensions. Incorporating additional necks on the body of a Helmholtz resonator (depending on whether they are open or closed) has been found to alter the resulting resonance frequency. For this study, tunable Helmholtz resonators to multiple resonance frequencies, are proposed and investigated utilizing additional necks. The resonance frequencies of various multi-neck Helmholtz resonators are first modeled with the use of the finite element method (FEM), then calculated with the use of an analytical approach and the results of the two approaches are finally compared. The results of this study show that Helmholtz resonators with multiple resonances at desired frequencies are achievable with the use of additional necks, while FEM and analytical methods can be used for the estimation of the resonance frequencies. Analytical and FEM approach results show a good agreement in cases of small number of additional necks, while the increasing differences in cases of higher neck additions, were attributed to the change in effective length of the necks as demonstrated by FEM. The proposed approach can be useful for tunable sound absorbers for room acoustics applications according to the needs of a space. Also, this approach can be applied in cases of additional tunable air resonances of acoustic instruments (e.g., string instruments).

## 1. Introduction

In architectural acoustics, the acoustic properties of the materials that make up a space largely determine its acoustic behavior. The sound absorption affects all the acoustic parameters which in turn define aspects of the acoustic behavior of a space [1]. In addition, for computational acoustics approaches, the knowledge of sound absorption of materials is necessary in order to model the acoustics of a space.

There are generally three categories of absorptive materials used in architectural acoustics applications [2]: porous absorbers, panel absorbers, and resonant absorbers. Porous absorbers are the most widely used due to their abundance of materials [3] (e.g., fiberglass, and mineral fiber products) and in turn also come in many categories [4]. Panel absorbers are usually nonporous lightweight sheets, with an air cavity behind them forming a resonant oscillating mass–spring system. They can be solid or perforated [5], and the air volume can be partly or completely filled with materials such as mineral wool or foam. Finally, resonant absorbers are usually Helmholtz resonators or similar enclosures, which are effective around their resonant frequency or partitions vibrating at their mass–air–mass resonance [2].

A Helmholtz resonator is a very important type of acoustic absorber with various applications in numerous fields. In general, a Helmholtz resonator is a container of gas (usually air) with a neck or an open hole. The compressible fluid inside the resonator acts as a spring [6], for the resonance frequency which depends on the volume of the resonator and the dimensions of the neck. The sound energy loss in a Helmholtz resonator occurs due to viscous loss at the wall of the neck, at the front wall of the resonator, at the edges of the neck and thermal loss in the resonator cavity [7]. Helmholtz resonators having a relatively small volume can be effective absorbers at low frequencies, unlike porous absorbers which are usually effective at medium and high frequencies [8]. This fact is very important, as the combination of these types of absorbers can cause a uniform absorption throughout the frequency range in a space.

Many architectural acoustics applications of the Helmholtz resonator can be found in the literature. Helmholtz resonators have been used as sound absorbers in churches [9,10,11] and also in ancient theatres [12]. They have been used also in the acoustic treatment of broadcasting studios [13] and conference rooms [14]. They have also been used to suppress specific eigenmodes in a room [15] and also to form a construction as a suspended ceiling [16]. Their sound absorption has been extensively studied [17,18] and various improvements have been proposed [19,20]. Also, various combinations with porous absorbers have been found to improve their acoustic performance [21]. Their sound absorption can be evaluated with appropriate sources [22,23] and excitation signals [24]. Besides architectural acoustics applications, various other applications exist for Helmholtz resonators such as in musical acoustics [25,26] or for sound speaker manufacturing [27]. Additionally, applications exist for acoustic energy harvesting [28], noise control in aircrafts [29] and gas turbines [30], and also as an acoustic metamaterial [31].

As presented before, in general, a Helmholtz resonator is a container of gas with an additional neck or opening. However, there are many variations regarding the shape of the neck (e.g., spiral [32,33], tapered [34], angled [35], petal shape [36]), or regarding the shape of the container [37,38]. An important and useful variation is the one where the Helmholtz resonator has more than one neck (usually called multi-neck Helmholtz resonators [39]). The result when you add more necks to the resonator body is that the resonance frequency changes which depends on the geometry and number of necks [40]. The same can be observed when there is a leak or a gap in the body of the resonator [40,41]; therefore, the understanding of the phenomenon is important for predicting the acoustic behavior of the resonator. For the numerical investigation of multi-neck Helmholtz resonators, various methods have been applied such as the boundary element method (BEM) [40] and finite element method (FEM) [42,43]. Among computational methods, FEM is probably the most widely used in the field of noise control [44], in architectural and environmental acoustics [45,46,47,48] and also in the frequency and the time domain [49,50,51].

This study set to propose and investigate a tunable Helmholtz resonator to multiple resonance frequencies, utilizing additional necks. For this reason, tunable multi-neck Helmholtz resonators are modeled with the use of FEM and also the resonance frequencies are calculated with an analytical formulation.

This paper has been organized as follows: Section 2 presents the methodology employed for the analytical and the FEM approach. Section 3 includes the findings of the research, while the discussion section analyzes the data, addresses the research question and identifies areas for further research. Finally, the conclusion section gives a brief summary of this research and contextualizes the study.

## 2. Methods

### 2.1. Theoretical Formulation (Single Neck, Multi-Neck)

#### 2.1.1. Single Neck

The resonance frequency of a single neck Helmholtz resonator can be calculated as follows [52]:

(1) $$f_{0}=\frac{c_{0}}{2\pi }\sqrt{\frac{\rho_{0}S_{1}}{V_{0}M_{1}}}$$

In the formula, *c*_0_ and *ρ*_0_ are the speed of sound and density of the air inside the body of the resonator, respectively. *V*_0_ is the cavity volume and *S*_1_ is the cross-sectional area of the neck. *M*_1_ is assumed to be the inertial mass of the air volume of the neck. For a cylindrical neck with diameter d_1_, and length$\;l_{1}$, the *M*_1_ can be estimated as $M_{1}=\rho_{0}\left(l_{1}+a_{1}d_{1}\right)$, where $a_{1}$ is the end correction coefficient which accounts for the ‘induced mass’ in the oscillatory flow in the vicinity of the two ends of the neck [53]. Values for the end correction can be found in the literature (e.g., circular aperture: π/4(≈0.785..) [54], unflanged pipe: 0.75 [55], flanged pipe: 0.85 [55]).

#### 2.1.2. Multi-Neck

A recent study [39] introduced a theoretical formulation for the calculation of the resonance frequency of a Helmholtz resonator with multiple necks. It was considered that the volume change inside the body of the resonator causes a compression of the fluid resulting in a pressure amplitude. Thus, pressure inside the cavity can be calculated with the use of the bulk modulus of the fluid according to [56]. An initial assumption for the determination of the analytical equation is that ‘p (pressure) can be assumed to be uniform inside the cavity and calculated with the bulk modulus of the fluid’. After a rigorous mathematical process, the following formula was proposed for the calculation of the resonance frequency of a multi-neck Helmholtz resonator:

(2) $$f_{0}=\frac{c_{0}}{2\pi }\sqrt{\frac{\rho_{0}}{V_{0}}\sum_{i=1}^{N}\frac{S_{i}}{M_{i}}}$$

For this formulation, a total of *N* necks are assumed to be connected to the cavity of the resonator, each neck *i*, with 1≤i≤N. *S_i_* is the cross-sectional area of each neck. Finally, as mentioned before, *M_i_* can be interpreted as the inertial mass of the neck air volume. In the case of cylindrical necks with diameter d_i_, as presented before, the neck air volume mass *M_i_* can be approximated as $M_{i}=\rho_{0}\left(l_{i}+a_{i}d_{i}\right)$. It should be also noted that Equation (2) can be expressed by introducing the resonance frequencies which correspond to the Helmholtz resonance frequencies with all, except the *i*-th, openings closed. Thus, Equation (2) becomes

(3) $$f_{i}=\frac{c_{0}}{2\pi }\sqrt{\frac{\rho_{0}}{V_{0}}\frac{S_{i}}{M_{i}}}$$

(4) $$f_{leaks}=\frac{c_{0}}{2\pi }\sqrt{\frac{\rho_{0}}{V_{0}}\sum_{i=2}^{N}\frac{S_{i}}{M_{i}}}$$

(5) $$f_{0}=\sqrt{\sum_{i=1}^{N}f_{i}^{2}}=\sqrt{f_{1}^{2}+f_{leaks}^{2}}$$

Equation (5) shows that the resonance frequency of a Helmholtz resonator with multiple necks is given by the square root of the sum of the squared resonance frequency without leaks $f_{1}^{2}$ and the squared resonance frequency with leaks only ($f_{leaks}^{2}$).

### 2.2. FEM Formulation and Setup

For the FEM formulation, the Helmholtz equation was used. In the equation, *p* is the acoustic pressure and *k* is the wave number. The equation is formed as:

(6) $$∆p\left(x\right)+k^{2}p\left(x\right)=0$$

According to the following equation, the normal derivative of the acoustic pressure p is associated with the normal fluid particle velocity $u_{f}$:

(7) $$u_{f}\left(x\right)=\frac{1}{i\omega \rho_{f}}\frac{\partial p(x)}{\partial n(x)}$$

In this equation $n\left(x\right)$ is the outward normal vector at a field point x and $\rho_{f}$ is the average density of the fluid.

As a next step, a weak formulation with weighting function χ(x) was used, and the equation is formed as follows [57]:

(8) $$\int_{\Omega }\chi \left(x\right)\left(∆p\left(x\right)+k^{2}p\left(x\right)\right)d\Omega \left(x\right)\;=\int_{\Gamma }\chi \left(x\right)i\omega \rho_{f}u_{f}\left(x\right)d\Gamma \left(x\right)+\int_{\Omega }\left(\nabla \chi \left(x\right)\nabla p\left(x\right)-k^{2}\chi \left(x\right)p\left(x\right)\right)d\Omega \left(x\right)=0$$

In this notation,Ω and Γ are the domain and boundary, respectively. Therefore, the acoustic pressure and particle velocity are shown as follows [49]:

(9) $$p\left(x\right)=\sum_{n=1}^{N}\Phi_{n}\left(x\right)p_{n}\;u_{f}\left(x\right)=\sum_{n=1}^{N}\Phi_{n}\left(x\right)u_{n}$$

Finally,$\Phi_{n}(x)$ is a basis function and $p_{n}$ and $u_{n}$ are the discrete acoustic pressure and fluid particle velocity at point x. Adding Equation (9) to Equation (8) [57],

(10) $$\left(K-k^{2}M\right)p=f$$

K and M are the stiffness and mass matrices. Vector f accounts for the source and vector and p accounts for the sound pressure values at the nodal locations.

For the FEM models, the software Comsol v.6 (Comsol Inc., Burlington, VT, USA) was used.. For the meshes, five elements per wavelength were applied, typical for similar FEM modeling [58]. For all FEM models and formulae, the speed of sound was set to 343 m/s and the density of air to *ρ* = 1.2 kg/m^3^. The domain was discretized with an unstructured mesh of quadratic Lagrange triangular elements. For the detection of the resonance frequencies, eigenvalue analysis was applied [46]. All the walls of the Helmholtz resonators were treated as Sound Hard Boundary. The Sound Hard Boundary (Wall) adds a boundary condition for a Sound Hard Boundary or wall, at which the normal component of the acceleration is zero. For this study, the viscothermal losses were not modeled. Since all the surfaces were considered as ‘Sound Hard Boundary’, there were no losses that occur in the acoustic thermal and viscous boundary layers near the walls. The main purpose of the research was to further investigate the issue of resonance frequency arising from the use of multiple necks. A following publication will focus on the issue of thermal and viscous losses for multi-neck Helmholtz resonators.

### 2.3. 3D Models

**Figure 1:**
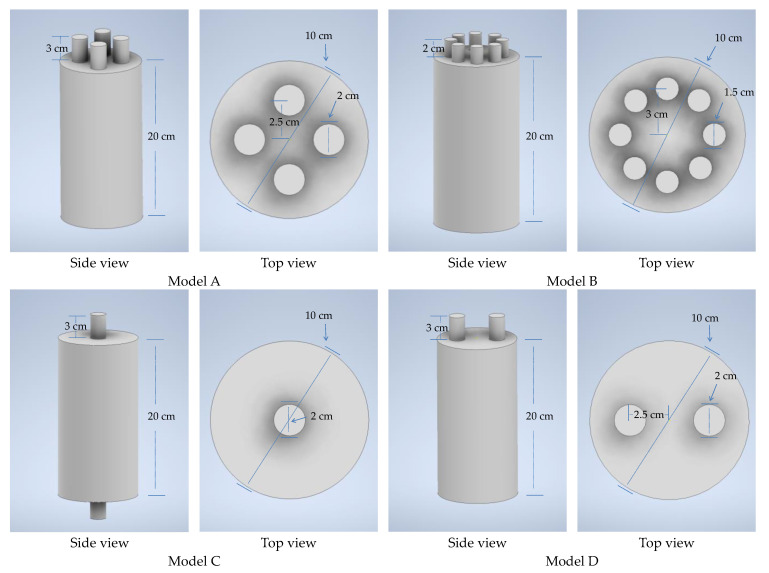
The 3D models (side and top view) of the multi-neck Helmholtz resonators (Models A–D).

For the modeling of the multi-neck Helmholtz resonators, the software Inventor v.2024 (Autodesk, San Francisco, CA, USA) was used. The shape and dimensions of the models are shown in Figure 1. Four different models were made, Model A and Model B, with 4 and 8 necks, respectively, and Models C and D with 2 necks.

**Figure 2:**
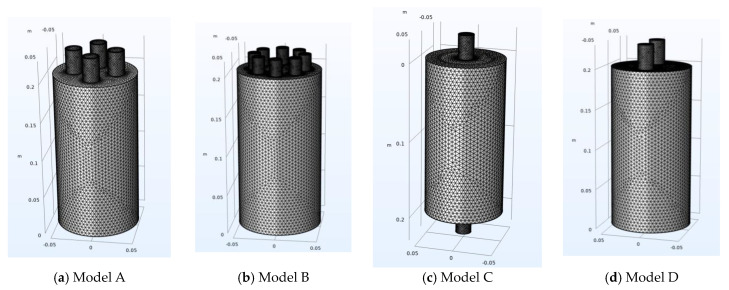
The 3D models with meshes of the multi-neck Helmholtz resonators (Models A–D).

Models C and D were chosen in order to have a comparison between resonators containing the same number of necks, placed in different positions on the body of the resonator. The rationale behind this selection is that, in this way, it can be investigated whether the different locations of the necks cause differences in the resonance frequencies. Models A and B were chosen to investigate and compare the effect of increasing number of necks on the resonance frequency in two similar but not identical models. In Figure 2, the 3D meshes for the models are presented.

## 3. Results

**Figure 3:**
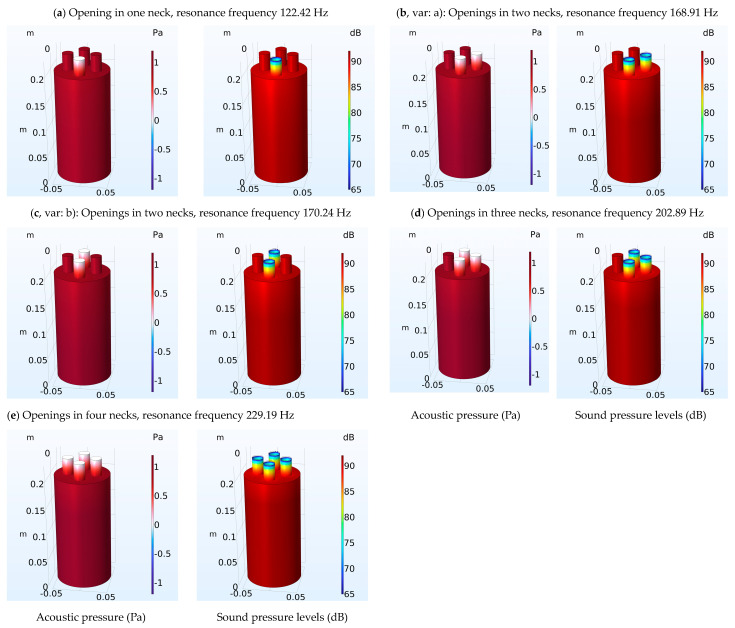
Helmholtz resonator (Model A): Acoustic pressure levels and sound pressure levels are presented for five different cases (**a**–**e**). In each case, there is an opening or openings in the top of the necks of the Helmholtz resonator. The necks with the openings can be distinguished by color differences in acoustic pressure and sound pressure levels in relation to the other necks. Cases b and c have same number of neck openings (2), but with different configurations.

**Figure 4:**
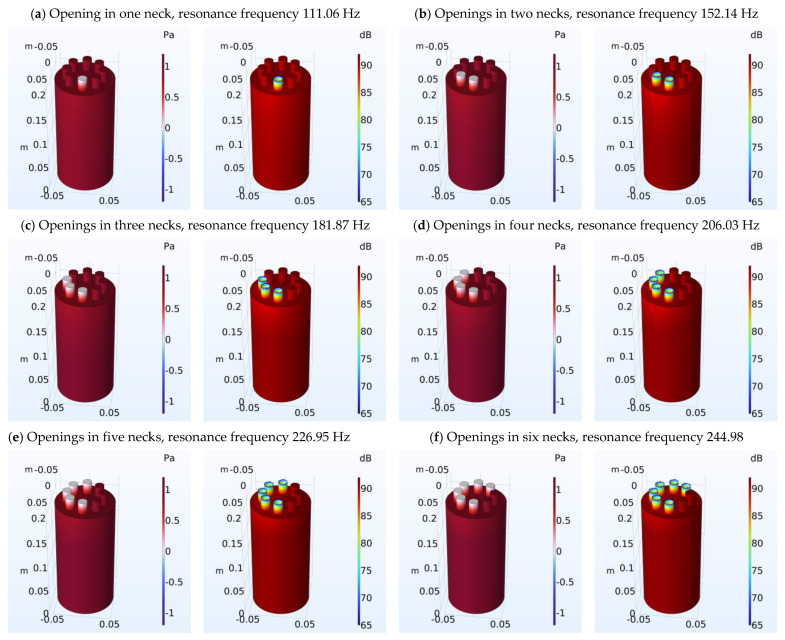

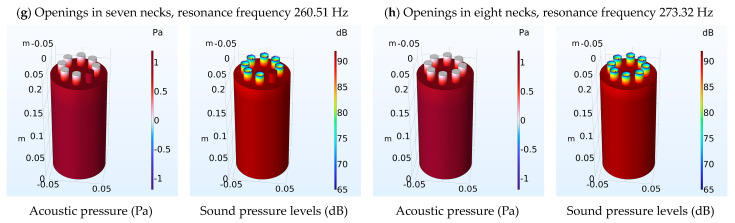
Helmholtz resonator (Model A): Acoustic pressure levels and sound pressure levels are presented in the Helmholtz resonator for eight different cases (**a**–**h**). In each case, there is an opening or openings in the top of the necks of the Helmholtz resonators. The necks with the openings in each case can be seen with the colored differences in acoustic pressure and sound pressure levels compared to the other necks.

Figure 3 and Figure 4 present the results of FEM modeling for Models A and B, respectively. For Model A (Figure 3), acoustic pressure levels and sound pressure levels are presented in the Helmholtz resonator (body and neck) for five different cases (a–e). In each case, there is an opening or openings in the top of the necks of the Helmholtz resonators. 

The necks with the openings in each case can be seen with the colored differences in acoustic pressure and sound pressure levels compared to the other necks. In cases b and c, two variations of the case with two openings in necks are presented (necks next to each other, and necks opposite each other). The reason these variations were studied is to investigate whether there are differences in the measured resonance frequencies.

Similarly for Model Β (Figure 4), acoustic pressure levels and sound pressure levels are presented in the Helmholtz resonator for eight different cases (a–h). In each case, there is an opening or openings in the top of the necks of the Helmholtz resonators. The necks with the openings in each case can be seen with the colored differences in acoustic pressure and sound pressure levels compared to the other necks.

In Table 1 and Table 2, a comparison of the results of the resonance frequency for Models A and B, respectively, is presented, calculated with the analytical approach and with FEM. The error of calculation is also presented.

### Multi-Neck Helmholtz Resonators with Same Number of Necks

As stated in methods section, in order to have a comparison between resonators containing the same number of necks, but necks placed in different places on the body of the resonator, the Models C and D were investigated. The body and necks geometry of both resonators are identical. The differences in the resonators are focused on the location of the necks. In Model C, the necks are on opposite surfaces, while in Model D, they are on the same surface. In Model C, the necks are in the center of the resonator, while in Model D, the necks are equidistant from the center of the surface.

**Figure 5:**
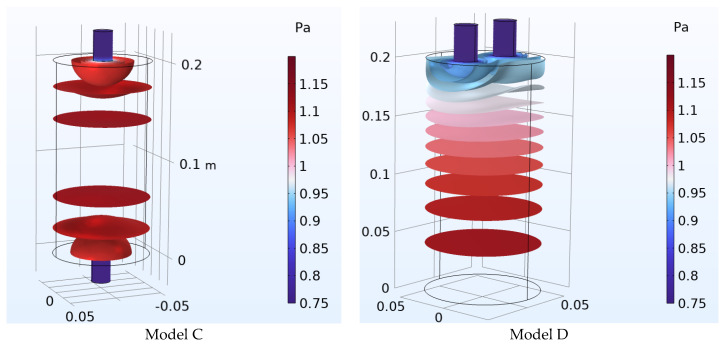
Helmholtz resonators (Models C and D): Acoustic pressure levels are presented in the Helmholtz resonators in the form of isosurfaces. In each case, there are openings in the top of the necks of the Helmholtz resonators. The same color legend was used for both models.

Figure 5 shows a comparison of the acoustic pressure distribution inside the resonators, in the case that both necks have openings. For the presentation of the acoustic pressure, isosurfaces were used so that the differences are more apparent. In addition, the same color legend was used for both cases.

**Figure 6:**
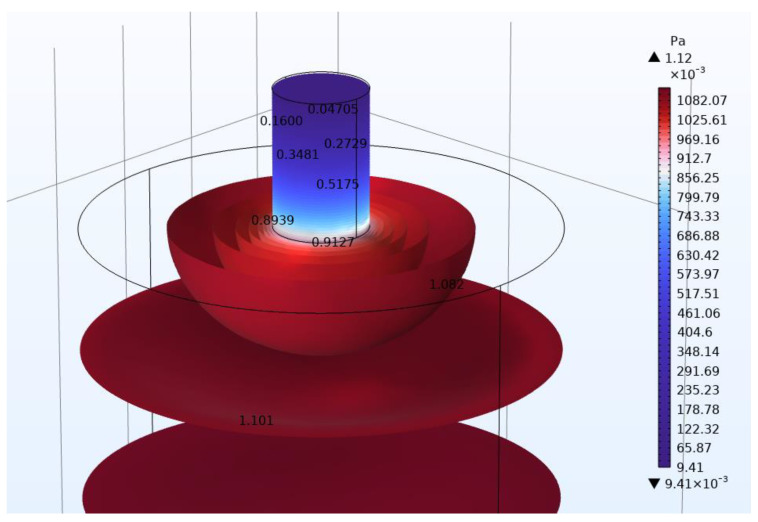
Helmholtz resonator (Model C): Acoustic pressure is presented in the form of isosurfaces. Openings are included in the top of both necks (bottom neck is not visible). Color legend range was chosen to make the differences in acoustic pressure more apparent.

**Figure 7:**
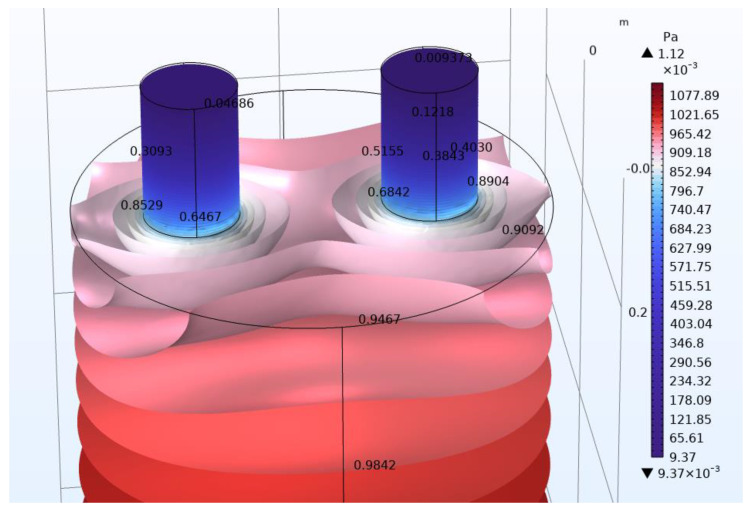
Helmholtz resonator (Model D): Acoustic pressure is presented in the form of isosurfaces. Color legend range was chosen to make the differences in acoustic pressure more apparent.

In Figure 6 and Figure 7, the acoustic pressures in the neck area of Models C and D, respectively, are presented, so that the differences could be more clearly visible. The acoustic pressure values are shown at points on the resonator and especially in the neck area. Again, for the presentation of the acoustic pressure, isosurfaces were used. Color legends were used to make the differences more apparent. It is evident that the acoustic pressure values in the neck area have differences in Figure 6 and Figure 7. For example, the differences are clearly seen at the base of the neck, where model C has a higher value (0.9127 Pa) than model D (0.7780 Pa). In Table 3 and Table 4, a comparison of the results of the resonance frequency for Models C and D, respectively, is presented, calculated with the analytical approach and with FEM. The error of calculation is also presented. All of the above will be discussed in the next section.

## 4. Discussion

The FEM modeling results for Models A and B were presented in Figure 3 and Figure 4 and also the results for the resonance frequency for the analytical method and FEM were presented in Table 1 and Table 2. The proposed Helmholtz resonators have resonance frequencies that are spaced apart by frequency differences that depend on the dimensions of the resonator necks. The different variations show that it is possible to make tunable Helmholtz resonators at any desired frequency. For example, to achieve small variations, suitably sized resonators (e.g., smaller neck diameter) can be added.

The FEM modeling results (acoustic pressure, and sound pressure level) for Models A and B are presented in Figure 3 and Figure 4. The figures show the acoustic pressure and sound pressure level distribution inside the resonator using color grading. It is evident that in the body of the resonator, the pressure has maximum values, while in the neck, the pressure has the smallest values. An important observation is that in the necks of the resonators which are closed, the pressure has a large value similar to the body of the resonator. During the analytical approach calculations, the volume of the necks with closed openings was considered as part of the body of the resonator.

Regarding the comparison of the results between the analytical approach and the FEM, there are several observations that can be made. In the initial case where a single neck is utilized in the Helmholtz resonator, the error of calculation is very small. This result is in agreement with previous studies (e.g., [59]) that have used FEM to estimate the resonance frequency of a resonator. In the case of multi-necks in the Helmholtz resonator, in general, the results show a good agreement between the analytical method and the FEM. It is evident that the resonance frequency increases with the addition of necks in the Helmholtz resonator, as has also been seen in related investigations [39,40]. A comparison of the results between FEM and analytical method shows small differences, especially for a small number of necks. This outcome is significant as the analytical method has been validated with the help of experimental data [39]. This in turn shows that the result of FEM modeling is most likely to correspond to experimental results. However, regarding a larger number of necks, there seems to be an increasing difference of the results between the analytical and the FEM formulation. This is discussed further in the next section.

### 4.1. Analytical and FEM Approach Differences

For Models A and B, regarding a larger number of necks, there seems to be an increasing difference of the results between the analytical and the FEM formulation. In order to explore further these results, Models C and D were investigated. As stated in methods section, for Models C and D, the body and necks geometry of both resonators are identical. The differences in the resonators are focused on the location of the necks. In Model C, the necks are on opposite surfaces, while in Model D, they are on the same surface. In Model C, the necks are in the center of the resonator, while in Model D, the necks are equidistant from the center of the surface. As can be seen in Table 3, the differences between analytical method and FEM for Model C are small. However, the differences between analytical method and FEM for model D, in the case of openings in both necks, seem to increase, similarly with Models A and B.

A possible explanation may be due to the initial assumptions in determining the analytical equation. It is stated that [39] ‘p (pressure) can be assumed to be uniform inside the cavity and calculated with the bulk modulus of the fluid’. However, it can be seen, especially in Figure 7 (Model D), that the pressure is not uniform inside of the cavity. Since the two necks (in Model D) are in close proximity, there seems to be an interaction in the acoustic pressure around the end of the necks resulting in a non-uniform acoustic pressure for Model D in that area compared to Model C. For Model C, in contrast, there is no interaction due to the necks being on opposite surfaces. The above shows us that the exact determination of the resonance frequency of a Helmholtz resonator depends on many parameters. The determination of all these parameters is beyond the scope of this text. In addition, a limitation of this research is that its conclusions need experimental validation so that they can be proven without any ambiguity. However, to further our research, we are planning to experimentally investigate the results of this study and also to determine all the parameters that affect the calculation of the resonance frequency. Further work needs to be carried out to establish the effectiveness of the method in a wide range of cases.

### 4.2. Possible Applications

The results of this study showed that it is possible to make tunable Helmholtz resonators at any desired frequency. For example, to achieve small variations, suitably sized resonators (e.g., smaller neck diameter) can be added. These tunable resonators could be used effectively in room acoustics or musical instrument design.

Especially for room acoustics applications, as mentioned in the introduction, such resonators can be used as absorbers in various applications [13,14,16]. Additionally, the ability of the resonators to be tunable makes them suitable for applications such as suppressing specific eigenmodes in a room [15]. Tunable Helmholtz resonators incorporating additional necks, have advantages over Helmholtz resonators (with one neck) currently commercially available. Helmholtz resonators in room acoustics are usually used as sound absorbers in the low-frequency range and are often placed in the corners for maximum absorption. Spaces, however, have different dimensions as a result of which the prevailing resonance frequencies in the low frequencies change. Therefore, having resonators that are highly customizable is an advantage for practical uses.

Regarding musical instrument design, tunable Helmholtz resonators may have some practical applications. The air cavity resonance of musical instruments is commonly called A0 resonance. It is sometimes called the “Helmholtz air resonance”; however, it is called so with a debate, since the A0 resonance has been found to not follow the typical formula of Helmholtz resonators [60]. In the majority of stringed instruments of the violin and the guitar family, and other instruments such as the harp and the ocarina, the air resonance is a vital part of the instruments’ sound. The body of these instruments usually includes an opening or openings in connection with the air cavity. The air inside the enclosed volume of the shell vibrates in and out through these openings. This resonance of the instruments is used to boost the sound of their lowest notes, which are often well below the frequencies of the lowest strongly excited, acoustically efficient, structural resonances. However, by adding additional necks to instruments with an air cavity and with the ability to open and close those necks, different frequency responses can be achieved of the instrument at will. This is likely to have many practical applications which have not been explored.

## 5. Conclusions

For this study, a tunable Helmholtz resonator utilizing multiple necks was proposed and investigated. Initially, the various resonance frequencies of a multi-neck Helmholtz resonator are first calculated with the use of a theoretical approach. In addition, the Helmholtz resonator is modeled with the use of the finite element method (FEM) and the results of the theoretical and the FEM modeling resonance frequencies are compared. The results of the two approaches show a very good agreement, with differences less than 1%.

This study demonstrated that with the utilization of appropriate number of necks, the Helmholtz resonator can be tunable to various desirable frequencies depending on the resonator’s geometry. The proposed approach can be useful for tunable sound absorbers for room acoustics applications according to the needs of a space. Also, this approach can be useful in cases of tunable air resonance of acoustic instruments.

## Figures and Tables

**Table 1:** Comparison of the results of the resonance frequency for Model A, calculated with the analytical approach and with FEM. Error of calculation is also presented. The positions of the necks with openings (2) in variations a and b can be seen in Figure 3.

Number of Necks	Resonant Frequency Analytical (Hz)	Resonant Frequency FEM (Hz)	Error of Calculation (%) Analytical-FEM
1	124.37	122.42	1.56
2 (var. a)	176.15	168.91	4.11
2 (var. b)	176.15	170.24	3.35
3	216.06	202.89	6.10
4	249.85	229.19	7.91

**Table 2:** Comparison of the results of the resonance frequency for Model B, calculated with the analytical approach and with FEM. Error of calculation is also presented.

Number of Necks	Resonant Frequency Analytical (Hz)	Resonant Frequency FEM (Hz)	Error of Calculation (%) Analytical-FEM
1	112.91	111.06	1.16
2	159.77	152.14	4.78
3	195.79	181.87	7.11
4	226.20	206.03	8.92
5	253.04	226.95	10.31
6	277.35	244.98	11.67
7	299.73	260.51	13.09
8	320.61	273.32	14.78

**Table 3:** Comparison of the results of the resonance frequency for Model C, calculated with the analytical approach and with FEM. Error of calculation is also presented.

Number of Necks	Resonant Frequency Analytical (Hz)	Resonant Frequency FEM (Hz)	Error of Calculation (%) Analytical-FEM
1	125.11	123.86	0.99
1	125.11	123.86	0.99
2	176.73	178.78	1.19

**Table 4:** Comparison of the results of the resonance frequency for Model D, calculated with the analytical approach and with FEM. Error of calculation is also presented.

Number of Necks	Resonant Frequency Analytical (Hz)	Resonant Frequency FEM (Hz)	Error of Calculation (%) Analytical-FEM
1	125.11	123.11	0.99
1	125.11	123.11	0.99
2	176.73	171.12	3.17

## Data Availability

The data presented in this study are available on request from the corresponding author.
